# Scanning Tunneling Microscopy Observation of Phonon Condensate

**DOI:** 10.1038/srep43214

**Published:** 2017-02-22

**Authors:** Igor Altfeder, Andrey A. Voevodin, Michael H. Check, Sarah M. Eichfeld, Joshua A. Robinson, Alexander V. Balatsky

**Affiliations:** 1 Nanoelectronic Materials Branch, Air Force Research Laboratory, Wright Patterson AFB, OH 45433, USA; 2 Department of Materials Science and Engineering, University of North Texas, Denton, Texas 76203, USA; 3 Department of Materials Science and Engineering and The Center for Two-Dimensional and Layered Materials, The Pennsylvania State University, University Park, PA 16802, USA; 4 Institute for Materials Science, Los Alamos National Laboratory, Los Alamos, NM 87545, USA; 5 Nordita, Center for Quantum Materials, KTH Royal Institute of Technology and Stockholm University, Roslagstullsbacken 23, 10691 Stockholm, Sweden

## Abstract

Using quantum tunneling of electrons into vibrating surface atoms, phonon oscillations can be observed on the atomic scale. Phonon interference patterns with unusually large signal amplitudes have been revealed by scanning tunneling microscopy in intercalated van der Waals heterostructures. Our results show that the effective radius of these phonon quasi-bound states, the real-space distribution of phonon standing wave amplitudes, the scattering phase shifts, and the nonlinear intermode coupling strongly depend on the presence of defect-induced scattering resonance. The observed coherence of these quasi-bound states most likely arises from phase- and frequency-synchronized dynamics of all phonon modes, and indicates the formation of many-body condensate of optical phonons around resonant defects. We found that increasing the strength of the scattering resonance causes the increase of the condensate droplet radius without affecting the condensate fraction inside it. The condensate can be observed at room temperature.

The concept of phonons describes quantum behavior and elementary excitations of sound waves in solids^1^,^2^,^3^,^4^,^5^,^6^,^7^,^8^,^9^,^10^,^11^,^12^,^13^,^14^,^15^,^16^,^17^,^18^,^19^,^20^. Phonons in layered two-dimensional (2D) materials have been extensively studied over recent years^1^,^2^,^3^,^4^,^5^ including low frequency interlayer vibrations^1^, flat optical bands with large lifetimes^2^,^3^,^4^, and thickness dependent anharmonicities^5^. The model system selected for this particular study is a defect-supported quasi-freestanding 2D monolayer, whose phonon oscillations can be directly probed by scanning tunneling microscope (STM)^20^, as shown schematically in Fig. 1a. Due to coherent scattering at defects, thermal phonons in such heterostructures can form spontaneous standing wave patterns resembling electronic Friedel oscillations in metals^7^,^21^. A significant difference stems from the fact that while for electrons only the density function 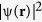 can be detected^21^; for phonons both the standing wave amplitude A(**r**) and the time factor *e*^iωt^ are observable characteristics^13^. In Fig. 1c we show the simulated interference wave packet for an interesting case of one-dimensional dispersionless (*ω*_k_ = *const*) optical phonons scattering at massive point defect. The construction of this curve involves summation of all standing wave modes across the 1^st^ Brillouin zone (BZ):


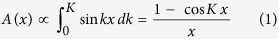


where *K* = *π*/*a* is BZ edge wave vector, and *a* is interatomic distance. The time-dynamics for all isofrequency modes in equation (1) is anticipated to be synchronized because of nonlinear intermode coupling^22^,^23^,^24^,^25^,^26^. The curve exhibits 2*a*-periodic nodes and 2*a*-periodic oscillation maxima decaying by *x*^−1^ law. Due to coherent nature of a wave packet, described by equation (1), and due to superposition of multiple phonon modes, the periodicity of interference maxima corresponds to wavelength cutoff (λ = 2*a*) at the BZ edge, not ½ λ as it usually occurs (see Fig. 1d). In a more applicable to our study 2D case, separation between neighboring atomic rows *a*_0_ determines the BZ edge wave vector *π*/*a*_0_; whereas equation (1), as we shall show in the Discussion part, describes a distribution of standing wave amplitude in orthogonal to these rows directions. In real 2D materials, due to formation of optical phonon bound states at defects^15^,^16^ and due to intermode phase and frequency synchronization^22^,^23^,^24^,^25^,^26^, the requirement of zero phonon bandwidth (Δ*ω* =0) most likely can be replaced with a softer requirement 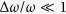. An interesting example of materials for experimental study of coherent optical phonon oscillations are semiconducting transition metal dichalcogenide (TMD) monolayers (ML)^1^,^2^,^3^,^4^,^5^ possessing an almost dispersionless homopolar optical branch ZO_2_ with estimated *∆ω*/*ω* ≈ 3%^2^,^3^ and large (~300 oscillations) phonon lifetimes^3^. Although in the absence of intermode coupling, the wave packets from Fig. 1c would only exist for *ω*/*∆ω*~30 oscillation periods, they may become essentially bound to defects when strong intermode coupling takes place. Apparently, for such transition to occur, the synchronization time has to be significantly shorter than both the optical phonon lifetime and the dephasing time *∆ω*^−1^. Synchronization of quasiparticles, and especially on the length scale ~λ, calls for analogy with Bose-Einstein condensation^27^,^28^, and the connection between these two effects was already shown in theory, on the examples of polaritons^27^ and magnons^28^.

## Results

The reported here STM measurements were performed on WSe_2_ layers grown by metal-organic chemical vapor deposition (MOCVD) method on top of graphene (see Methods). The STM studies revealed phonon standing wave patterns only on quasi-freestanding 1 ML islands, like a triangular shaped island presented in Fig. 2a. On STM cross-sections a quasi-freestanding geometry of WSe_2_ nanostructures manifests as additional 2.5 Å elevation of the first atomic layer. As we show in Fig. 2c, measured by STM heights are 6.4 Å for some of 1 ML islands (dashed line), whereas for other 1 ML islands measured heights are 8.9 Å (solid line). For WSe_2_ pyramids^29^, also present at the surfaces of our samples (see Fig. 2b and d), STM cross-sections typically reveal 6.5 Å layer heights, which is close to bulk value; although in some instances the bottom layers in pyramids can also be elevated to 8.9 Å, as shown in Fig. 2e. The additional 2.5 Å elevation of the first atomic layer was observed in more than 50% of nanostructures in our samples. As we discuss in Fig. 2f, these observations indicate that a significant portion of WSe_2_ nanostructures in our samples possesses quasi-freestanding geometry^4^,^30^ being supported by intercalation defects produced during sample growth.

**Figure 1:**
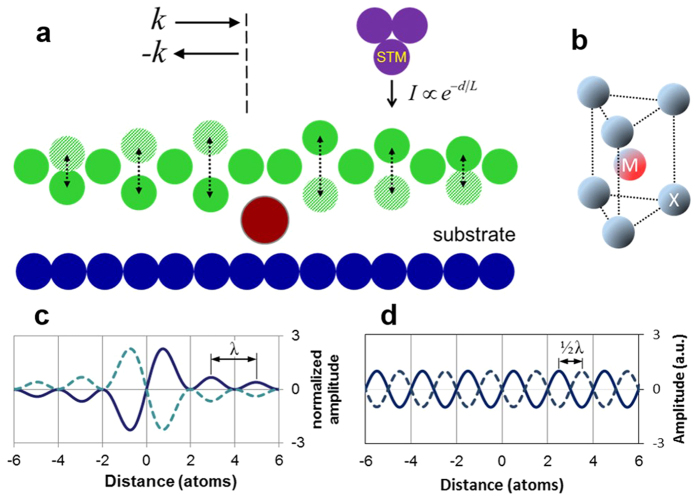
STM-based optical phonon detection setup. (**a**) Schematic illustration of phonon interference in a heterostructure comprising: substrate, intercalation defect, and top layer (whose unit cell structure, for simplicity, is not shown; see also (**b**)). Because the defect is attached at high-coordination interatomic site, the reflection points for phonons are shown laterally offset. Vertical dotted arrows indicate out-of-plane atomic oscillations. Due to sharp nonlinear dependence of tunneling current (*I*) on vacuum gap (*d*) these oscillations can be detected (rectified) by STM^20^. (**b**) Unit cell of 1H-MX_2_ (transition metal dichalcogenide) monolayer^1^,^2^,^3^,^4^,^5^. In our experimental setup, the upper plane of chalcogen atoms is used for STM monitoring optical phonon oscillations, whereas the lower plane is used for coupling to intercalation defects. (**c**) Simulated interference pattern for dispersionless 1D optical phonons phase-synchronized due to intermode coupling. The vertical axis of this plot is normalized on typical phonon amplitude and because of oscillations is shown to periodically reverse its sign. The defect is assumed point-like at *x* = 0. (**d**) Distribution of amplitudes for a standing wave produced by BZ edge state only: *A*(*x*) ∝ sin *Kx*. Due to defect-imposed boundary condition, all interference maxima occur at interatomic sites, and such standing wave is not observable.

**Figure 2:**
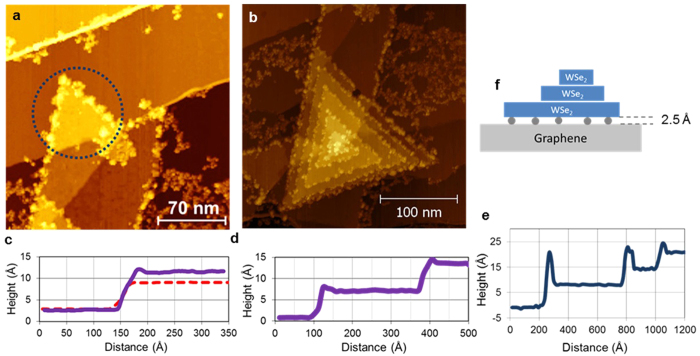
STM images and cross-sections of WSe_2_ nanostructures. (**a**) The dotted line on this STM image surrounds a triangular shaped 1 ML WSe_2_ island. (**b**) STM image of WSe_2_ spiral pyramid. The particles observed at the edges of WSe_2_ domains in (**a**,**b**) are the tungsten oxide phase^59^,^60^. STM measurements of phonon standing waves were performed outside of the edge areas. (**c**) STM cross-sections of 1 ML islands reveal 6.4 Å and 8.9 Å layer heights. (**d**) STM cross-section of a spiral pyramid reveals 6.5 Å layer heights. (**e**) STM cross-section of the elevated WSe_2_ pyramid. Only the first atomic layer is elevated to 8.9 Å, the upper layers have normal 6.5 Å heights. (**f**) A significant portion of WSe_2_ nanostructures in our samples possesses quasi-freestanding geometry being supported by intercalation defects produced during sample growth. A similar effect was earlier reported in ref. 4.

**Figure 3:**
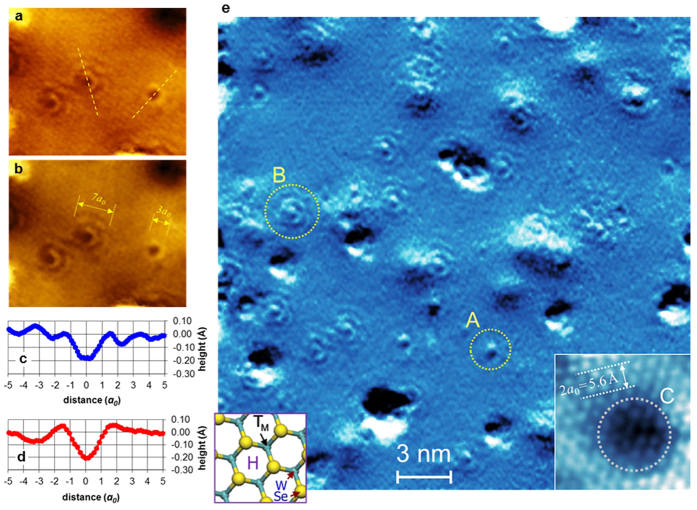
STM images of quasi-freestanding WSe_2_ islands. (**a**) STM image of 90 × 75 Å^2^ area of elevated 1 ML island obtained at 2 V sample bias. (**b**) Image of the same area at 3 V bias. (**c**) Cross-section of type-B (multi-ring) pattern from (**a**). (**d**) Cross-section of type-A (single-ring) pattern from (**a**). The STM cross-sections are oriented perpendicular to atomic rows, and the horizontal axes are normalized to *a*_0_. The central minima in (**c**,**d**) have slightly different shapes due to different contributions of *cosine* modes (see Discussion part and Supplementary Note 2). (**e**) The larger scale, 260 × 260 Å^2^, STM image of phonon interference patterns on elevated 1 ML island. The image uses gradient contrast. One of type-B and one of type-A patterns are schematically surrounded by dotted lines. For gradient contrast, the missing half-rings are less visible. Bright-contrast features originate from residual contaminating particles. (**Left inset**) The left inset shows different absorption sites for defects, H-site vs. T_M_-site, that may also cause type-A vs. type-B standing wave patterns. (**Right inset**) STM image in the right inset clarifies the horizontal axis units in (**c**,**d**) and the orientation of crystal axes in (**a**,**b**,**e**). The pattern on this STM image (surrounded by dotted line type-C pattern) only contains a broad central minimum.

The room temperature STM measurements on quasi-freestanding 1 ML WSe_2_ islands are presented in Fig. 3. The STM image in Fig. 3a shows the 90 × 75 Å^2^ fragment of the surface of such island. The image was obtained at 2 V tunneling bias and 35 pA tunneling current. The interference rings in this image can be clearly observed. On the right side of the image, one can see a single-ring pattern (later in the text: type-A pattern) with a diameter of ≈8.2 Å. On the left side of the image, we observe several multi-ring (type-B) patterns. For this type, the first ring also has a diameter of ≈8.2 Å; the second ring has a diameter of ≈19.4 Å; and the fragments of the third interference ring can also be seen in some cases. The STM cross-sections of type-B and type-A patterns are shown in Fig. 3c,d. The cross-sections are oriented perpendicular to atomic rows, and their horizontal axes are normalized on *a*_0_ (*a*_0_ = 2.8 Å^31^, see the right inset in Fig. 3e). The central minima have 0.2 Å depths, and a typical height of the first interference maxima is 0.1 Å. The absence of surface adatoms or other visible defects in the centers of these patterns indicates that the interference is induced by subsurface defects. The ring diameters correspond to 3*a*_0_ and 7*a*_0_ in exact accordance with Fig. 1a prediction for intercalation defects. As it follows from the comparison of Fig. 1a and c, because intercalating atoms are attached at high-coordination interatomic sites, the “reflection points” for phonons become ±½ *a*_0_ shifted, increasing, thus, the interference ring diameters from Fig. 1c predicted {2, 6} to experimentally observed {3, 7}. As we show in Fig. 3b, the ring diameters do not depend on the bias voltage, and the separation between interference maxima always corresponds to 2*a*_0_. Please note: a closer look at type-B patterns reveals visible angular segmentation that will be later discussed in more detail.

In Fig. 3e we show a larger scale view of phonon standing wave patterns. The size of this STM image is 260 × 260 Å^2^. To enhance the visibility of interference patterns the image was differentiated along the vertical (scan) axis. From this image, we conclude that type-B patterns appear in 85% of all observed cases. From the total number of interference patterns in Fig. 3e, knowing the total number of WSe_2_ unit cells, we find that the density of intercalation defects significantly contributing to phonon scattering is ≈0.3%. It seems very likely that the two types of interference patterns on STM images are associated with two different kinds of boundary conditions, decay laws, and scattering regimes^32^ imposed by intercalation defects. The observed difference could be induced by two types of intercalating molecules with different masses, for example CO vs. H_2_O molecules, or it could be caused by two preferential attachment sites for intercalation defects^33^,^34^ (see left inset of Fig. 3e). The right inset of Fig. 3e demonstrates another type of observed patterns, the type-C pattern possessing only a broad central minimum and originating most likely from weakly scattering defects and/or interfacial charges. The type-C patterns are unnoticeable on gradient-contrast Fig. 3e. The total density of intercalation defects is apparently more than 0.3%.

**Figure 4:**
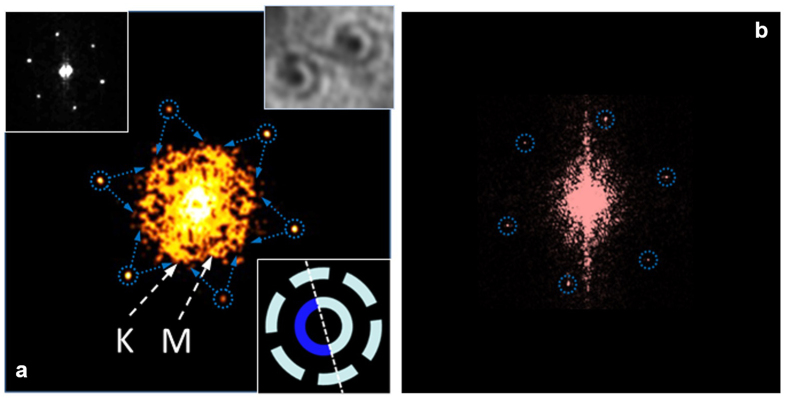
STM Fourier transforms. (**a**) STM Fourier map for elevated monolayer island shown in Fig. 3 reveals 2D hexagonal BZ. The 1^st^ BZ in this Figure is constructed from the intersection of six Bragg planes schematically shown as six pairs of facing each other arrows. The BZ symmetry points K and M are also indicated by arrows. (**Lower inset**) The lower inset shows a schematic view of angular segmentation of interference rings for type-B pattern. Suppressed interference maxima are shown as darker. Dashed line corresponds to STM cross-section from Fig. 3c. For such patterns, the real-space distribution of phonon amplitudes represents a superposition of three quasi-one-dimensional oscillations directed perpendicular to atomic rows. (**Upper-left inset**) STM Fourier map for elevated monolayer island at 100 K does not reveal any interference related features. (**Upper-right inset**) The inset shows the experimentally observed angular segmentation of interference rings for type-B patterns after readjustment of local image contrast. (**b**) STM Fourier map for non-elevated monolayer island at room temperature does not reveal any interference related features.

The interference patterns were only observed on elevated monolayer islands. In order to illustrate this, in Fig. 4 we show a comparison of 2D STM Fourier maps corresponding to elevated (Fig. 4a) and non-elevated (Fig. 4b) monolayer islands. The six peaks from the hexagonal crystal lattice in Figs. 4ab represent the reciprocal lattice vectors ±2π/*a*_0_. In Fig. 4a, the 1^st^ BZ is constructed from the intersection of six Bragg planes. The hexagonal disk in Fig. 4a represents the area of *k*-space that contributes to interference. Essentially, Fig. 4a confirms that the interference patterns are produced by superposition of all optical phonon modes from the 1^st^ BZ. The concept of BZ plays important role in condensed matter physics, and STM imaging optical phonon quasi-bound states represents an interesting method of its direct observation. Because for optical phonons an interference pattern represents a spatial distribution of a standing wave amplitude, each *k*-point inside the 1^st^ BZ corresponds to the same *k*-point of the Fourier map, not 2*k* as it occurs for electrons^35^. The absence of interference-related features in Fig. 4b confirms that the standing waves are produced by phonon scattering at subsurface defects. The interference signals in Fig. 4a primarily originate from type-B patterns, because only for this type the pattern periodicity can be defined. The hexagonal symmetry of the Fourier image in Fig. 4a indicates that phonon wave packets may also possess hexagonal real-space symmetry. Indeed, the analysis of Fig. 3a,b shows that for type-B patterns a visible angular segmentation of the interference amplitude takes place. The outer interference rings on these images reveal visible 60° segmentation relevant to hexagonal symmetry. Such angular segmentation indicates that real-space distribution of phonon amplitudes for type-B patterns represents a superposition of three quasi-one-dimensional oscillations directed perpendicular to atomic rows. The inner interference rings show visible 180° segmentation, indicating the symmetry breaking. Schematic view of these segmentations is shown in the lower inset of Fig. 4a. The experimentally observed ring segmentation can also be seen in the upper-right inset of Fig. 4a obtained after image contrast enhancement. For type-A patterns, a similar symmetry breaking effect can also be observed upon significant readjustment of the image contrast. The possibility of complete or partial suppression of certain interference maxima represents a unique feature of phonon standing waves described by equation (1). Such suppression, for example, may occur due to formation of coherent and incoherent spatial domains predicted by phase-synchronization theories^22^,^23^. The locations of the suppressed interference maxima are most likely determined by boundary conditions imposed by defect and are anticipated to be reproducible for a given type of interference pattern, in excellent agreement with our experimental results. At lower temperatures, the pattern contrast decreases, most likely due to decrease of phonon amplitudes and less efficient synchronization (see Supplementary Figure 3), and at 100 K the interference cannot be observed (see the upper-left inset of Fig. 4a).

## Discussion

**Figure 5:**
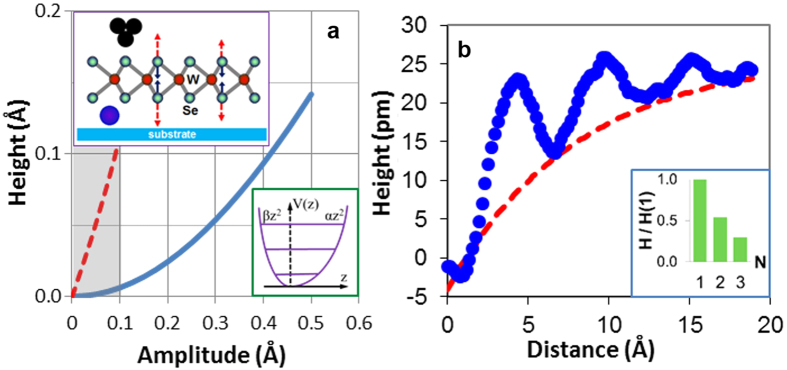
Dependence of STM signals (*h*) on phonon amplitudes (*A*). (**a**) The solid curve shows expected *h*(*A*) dependence for (symmetric) harmonic atomic motion. The dashed line shows expected *h*(*A*) dependence for factor of two “apparent” asymmetry of outward vs. inward atomic motions. Grayed area indicates the anticipated range of phonon amplitudes. (**Upper inset**) The upper inset illustrates atomic motion for mirror-symmetric out-of-plane ZO_2_ oscillations under phonon interference conditions. Dashed vs. solid arrows indicate the “apparent” asymmetry of atomic motion detected by STM. (**Lower inset**) The lower inset shows the energy levels for asymmetric harmonic oscillator. The dashed line in (**a**) corresponds to (*β*/*α*)^1/2^ = 2. (**b**) Cross-section of type-B pattern. After subtraction of the background, indicated by a dashed curve, the profile of type-B pattern resembles simulated plot from Fig. 1c. (**Inset**) The inset shows typical STM heights (normalized) of first three interference maxima for type-B patterns. The observed 1/*x* decay law also confirms linear *h*(*A*) dependence for studied samples.

All our experimental findings can be successfully explained by formation of coherent phonon quasi-bound states around intercalation defects. The most likely source of these STM signals is nearly dispersionless out-of-plane optical phonon branch ZO_2_ 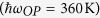, connected to Raman peak 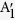 at 250 cm^−1^, whose atomic motion represents mirror-symmetric oscillations of Se atoms, as illustrated in the upper inset of Fig. 5a. For this phonon branch, the simulated dependences of topographic STM signals (*h*) on phonon amplitudes (*A*) are presented in Fig. 5a for symmetric (solid curve) and asymmetric (dashed curve) harmonic atomic motions. The construction of these curves takes into account high-frequency oscillations of a vacuum gap (see Fig. 1a) and exponential distance dependence of tunneling current with decay length *L* = 0.4 Å established for combination of studied islands and STM tip. For simulation of a dashed curve in Fig. 5a, a factor two outward vs. inward motion asymmetry has been assumed (see lower inset of Fig. 5a). Although optical phonon oscillations are too fast to be detected by STM in real-time, the increase of average tunneling current takes place due to these oscillations forcing the STM tip to retract from the surface. We found that for symmetric harmonic oscillations *h*(*A*) ∝ *A*^2^/*L*, whereas for asymmetric oscillations *h*(*A*) ∝ *A*. Typical room temperature phonon amplitude (oscillation amplitude for each Se atom), estimated for ZO_2_ branch using quantum harmonic oscillator model, is 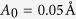 (5 pm) from which about ⅔ is due to zero-point motion. The peak amplitude anticipated for first interference maxima is ≈2 times larger, *A*_max_ ≈ 10 pm (see Fig. 1c). The expected range of phonon amplitudes is indicated as vertical grayed area in Fig. 5a. The comparison of curves in Fig. 5a clearly shows that in order to justify the experimentally observed ~10 pm STM signals, a significant apparent oscillation asymmetry has to be present in the studied system. As it was noticed in ref. 36, asymmetric harmonic oscillators can develop due to dipole-like interactions. Because their energy levels (see lower inset of Fig. 5a) are also equidistant, this type of “anharmonicity” would be unnoticeable in the temperature dependence of Raman shifts^5^. Generally, all types of atomic motions resulting in asymmetric probability distribution for oscillator are anticipated to produce linear *h*(*A*) dependence. We also cannot exclude the possibility that the “apparent” oscillation asymmetry is being enhanced by tunneling measurements due to asymmetric electronic response associated with periodic stretching and compression of W-Se bonds^37^,^38^. In general, the visibility of surface phonon oscillations for STM is anticipated to be enhanced due to phonon-mediated deformation potential modulating the electronic structure. Because the spatial radius of optical phonon deformation potential is comparable to unit cell size (≈3 Å), observed by STM patterns resemble slightly smoothed segmented rings. In view of the important role of deformation potential for STM detection of surface atomic oscillations, the contribution of another weakly dispersive, in-plane polarized LO_2_ branch^2^,^3^ also cannot be ruled out.

To better understand the difference between the two observed types of standing wave patterns, one should take into account that for optical phonons the scattering centers can develop due to local change of the oscillation frequency in the presence of intercalation defects. For example, for ZO_2_ phonons local oscillation frequency introduced by molecular adsorption at the most favorable *H*-site^33^ can be estimated (see Supplementary Note 1) from the increase of the oscillating mass


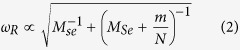


where *M*_*Se*_ is a mass of selenium atom, *m* is a mass of adsorbed molecule, and *N* = 3 is a coordination number for *H*-site. The corresponding frequency shift, normalized to phonon frequency *ω*, is given by


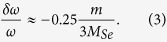


**Figure 6:**
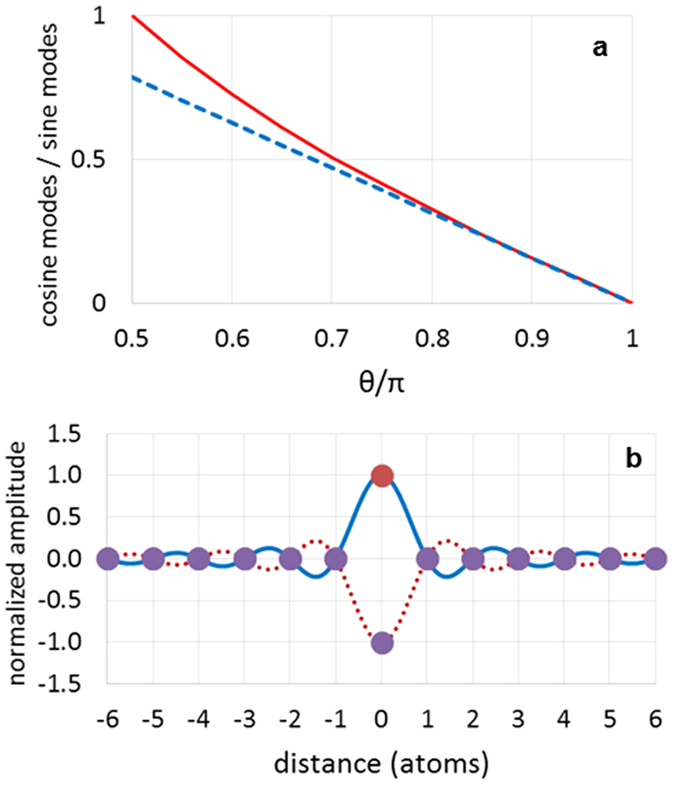
Analysis of the contribution of *cosine* modes to standing wave patterns. (**a**) The solid curve shows the amplitude ratio of *cosine* and *sine* modes in the interval 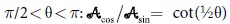 (see Supplementary Note 2). Except for near-resonance region around π/2, this curve can be approximated by linear dependence 0.5(*π* − θ), which is shown as dashed line. (**b**) The solid curve shows the wave packet obtained by coherent superposition of all *cosine* modes across the BZ, similar to equation (1): *A*(*x*) ∝ *x*^−1^ sin *Kx*. The vertical axis of this plot is normalized on typical phonon amplitude and because of oscillations is shown to periodically reverse its sign. The solid circles on top of these curves indicate the amplitude values at atomic positions. This type of standing wave is essentially undetectable, except for at x = 0. For type-B pattern, the corresponding feature is manifested as ~3 pm flattening of the cross-sectional curve at defect location that can be observed in Fig. 3c. For type-A patterns, such evident “zero” features were not found because of less resonant scattering conditions. The oscillation amplitude for this feature (*A*_*z*_) is related to other experimentally measured parameters: *A*_*z*_/(½ *A*_max_) ≈ 0.5(*π* − θ). The experimental observation of “zero” feature directly confirms the proposed resonant phonon scattering mechanism.

For H_2_O adsorption we find *δω*/*ω* = −2%, for CO adsorption *δω*/*ω* = −3%. The frequency shifts are small and comparable to intrinsic phonon linewidth *∆ω*. The distribution of phonon scattering cross-sections σ and scattering phase shifts θ for such resonant defects can be established from the scattering theory^32^,^39^,^40^ (see Supplementary Note 2)


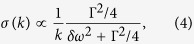



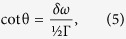


where *δω* = *ω*_*R*_ − *ω* is determined by equation (3) is full width at half maximum (FWHM) of the defect-induced local resonance, and *k* is phonon wave vector. Equation (4) represents the 2D analogue of the Breit-Wigner formula^40^. For negative values of *δω*, the scattering phase shift belongs to the interval *π*/2 < θ < *π*, and the dominant contribution to standing wave patterns arises from *sine* modes, as in equation (1). We also find that at these conditions *σ*(*k*) ∝ *k*^−1^, i.e. resonant scattering cross-section of 2D phonon is proportional to its wavelength. As a result, in two dimensions the ∝*k* increase of the phase space area is compensated by *k*^−1^ dependence of phonon scattering probabilities. Consequently, for type-B patterns, most frequently observed on STM images, real-space distribution of phonon amplitudes represents a superposition of three (120° rotated) quasi-one-dimensional oscillations described by equation (1). In Fig. 5b and in the inset of Fig. 5b we show the profile of type-B interference pattern and experimentally measured height distribution of interference maxima for such patterns. The fact that the interference maxima decay by *x*^−1^ law confirms our earlier conclusion that real *h*(*A*) dependence in our samples is linear. After subtraction of the background, the cross-sectional profile in Fig. 5b strongly resembles 1D simulation from Fig. 1c, confirming that optical phonons in our samples form synchronized coherent superposition. The modeling simulations have shown that the background curve in Fig. 5b appears to be the result of quadratic “superposition” of all phonon modes (see Supplementary Fig. 1), possibly indicating that phonon quasi-bound states possess both coherent and *incoherent* components. Phase and frequency synchronization of phonons was earlier discussed in theory for term crossing^24^,^25^, where it occurs due to anharmonic intermode coupling. In our samples, superposition involves phonon states with incommensurate wave vectors, and their coupling primarily manifests near defects. For type-B patterns, this corresponds to *R*_*B*_ ≈ 1.5 nm radius regions, most likely due to enhanced intermode coupling associated with resonant defects. There are several reasons that can justify the resonant enhancement of phonon-phonon interactions at such defects: (a) higher scattering cross-sections and correspondingly higher probabilities of “finding” a scattered phonon inside such defects^31^,^39^,^40^, (b) non-vanishing oscillation amplitudes at resonant defects owing to additional to equation (1) contribution of *cosine* modes (see Fig. 6a). For standing wave packets produced by coherent superposition of *cosine* modes, oscillation amplitudes follow *x*^−1^ sin *Kx* dependence and vanish at all atomic sites, except for defect location. As we show in Fig. 6a,b, the amplitude associated with this “zero” feature can be estimated in convenient for data analysis terms


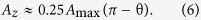


On STM cross-sections, this feature is manifested as ~3 pm flattening of the central interference minima, which can also be observed in Figs 3c and 5b. For type-A patterns, such evident “zero” features were not found most likely because of less resonant scattering conditions. From this data, we find that θ_A_ − θ_B_ ≈ 40°. This method for estimating phonon scattering phase shifts is different from the methods earlier used for electrons^21^,^41^. Only the first interference maximum from equation (1) can be observed for type-A patterns, indicating a smaller *R*_*A*_ ≈ 0.7 nm effective radius of quasi-bound states for such defects.

**Figure 7:**
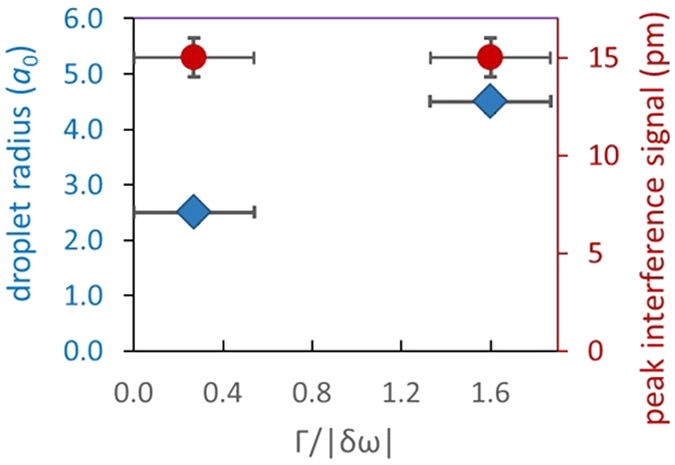
Analysis of condensate droplets. The room temperature dependence of peak interference signal on STM images (red circles) and estimated droplet radius (blue diamonds) on the “strength” 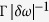 of the scattering resonance. The 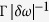 values were estimated from the experimentally measured height of “zero” feature (error bar ±0.5 pm) using equations (5) and (6), i.e. Γ|*δω*|^−1^ ≈ 8*A*_*z*/_*A*_*max*_. For type-A pattern, the height of “zero” feature was assumed at STM signal sensitivity limit of 0.5 pm, although its actual value is most likely larger. For less resonant type-A defects the droplet radius decreases, whereas the condensate fraction (directly related to interference signals) remains unchanged. The peak interference signals in this Figure represent the height of the 1^st^ interference maxima after background subtraction. The effective radius is estimated as 2.5*a*_0_ for type-A and 4.5*a*_0_ for type-B droplets, according to locations of 1^st^ and 2^nd^ interference minima.

Since the typical timescale of our STM experiment exceeds both the lifetime and the dephasing time of optical phonons by at least 12 orders of magnitude, a many-body condensation^27^,^28^,^42^,^43^,^44^,^45^,^46^,^47^,^48^,^49^,^50^,^51^,^52^,^53^,^54^,^55^,^56^,^57^,^58^ mechanism capable to synchronize all thermal phonons within ~1 oscillation period is required for their interference patterns to be observable using STM. Synchronization of phonons essentially implies that instead of each of them possessing an independent oscillation phase φ_k_(t), all φ_k_(t) become equal to Φ(t) (condensate phase), the behavior well known earlier for systems of coupled mechanical oscillators^42^ and for spin precession in cold vapors^51^. Due to finite lifetime of optical phonons, the content of the condensate is anticipated to be dynamically renewed, and all newly emerging phonons around the defects are anticipated to join the condensate droplet being affected by its collective oscillation field *e*^iΦ(t)^. At low temperatures, the coherent collective oscillation field most likely disappears when the average number of thermal phonons inside the interference area decreases to ~1. This can explain the disappearance of interference signals at low temperatures in Fig. 4a (see Supplementary Figure 4). The comparison of type-A and type-B condensate droplets in Fig. 7 clearly indicates that increasing the strength of the scattering resonance causes the increase of the effective radius without affecting the condensate fractions inside the droplets.

We anticipate that defect-mediated phonon condensates can become useful components of quantum computers. The observed effects may also be important for understanding the room temperature coherence mechanism of biological systems^53^. Although strong anharmonicity can explain large observed interference signal amplitudes, the possibility of unusually high phonon populations inside the droplets anticipated for real-space Bose-Einstein condensate^57^,^58^ also cannot be excluded.

## Methods

The WSe_2_ films for our study were prepared using MOCVD technique on top of several layers of epitaxial graphene on SiC(0001) substrate. The growth details, including preliminary Raman, TEM, and XPS characterization, were described in the earlier publications^59^,^60^. The STM measurements were performed using ultra-high vacuum system UHV300 from RHK Technology, with base pressure 7 × 10^−11^ Torr. Before STM measurements, the samples were *in situ* annealed to 350 °C for few hours in order to eliminate the adsorbed water from their surfaces. The sample temperature was estimated using K-type thermocouple. For STM measurements, we used commercial Pt-Ir STM tips from Bruker Corp. that were *in situ* cleaned using electron beam heating technique.

## Additional Information

**How to cite this article**: Altfeder, I. *et al*. Scanning Tunneling Microscopy Observation of Phonon Condensate. *Sci. Rep.* **7**, 43214; doi: 10.1038/srep43214 (2017).

**Publisher's note:** Springer Nature remains neutral with regard to jurisdictional claims in published maps and institutional affiliations.

## Supplementary Material

Supplementary Material
