# Supplementary material for: Scanning Tunneling Microscopy Observation of Phonon Condensate
Source: Sci Rep. 2017 Feb 22;7:43214. doi: 10.1038/srep43214 (PMC5320553; doi:10.1038/srep43214)
Supplement: Supplementary Material [file srep43214-s1.pdf]

Supplemental Material for manuscript

Scanning Tunneling Microscopy Observation of Phonon Condensate

Igor Altfeder[1], Andrey A. Voevodin[1,2], Michael H. Check[1], Sarah M. Eichfeld[3], Joshua A. Robinson[3] and Alexander V. Balatsky[4,5]

[1]Nanoelectronic Materials Branch, Air Force Research Laboratory, Wright Patterson AFB, OH 45433, USA

[2]Department of Materials Science and Engineering, University of North Texas, Denton, Texas 76203, USA

[3]Department of Materials Science and Engineering and The Center for Two-Dimensional and Layered Materials, The Pennsylvania State University, University Park, PA 16802, USA

[4]Institute for Materials Science, Los Alamos National Laboratory, Los Alamos, NM 87545, USA

[5]Nordita, Center for Quantum Materials, KTH Royal Institute of Technology and Stockholm University, Roslagstullsbacken 23, 10691 Stockholm, Sweden

Supplementary Note 1: Discussion about a frequency shift introduced by adsorbed molecule.
Supplementary Note 2: Discussion about scattering phase shifts and relative contribution of *cosine* modes.
Supplementary Figure 1: Simulation of the background curve.
Supplementary Note 3: Discussion about phonon populations.
Supplementary Figure 2: Tunneling density of states for studied islands.
Supplementary Figure 3: Temperature dependence of interference signals.
Supplementary Figure 4: Temperature dependence of average phonon numbers.

Supplementary Note 1.

This result can be obtained from the simple analogy with symmetric linear triatomic molecule, whose one side was loaded with a small additional mass, so that $\delta m/m << 1$. In the linear (proportional to δm) approximation, the new oscillation frequency depends only on harmonic mean value of two opposite oscillating masses, whereas the central mass does not affect the final result.

Supplementary Note 2.

According to scattering theory, the scattering phase shift θ=0 when reflection occurs from less dense media; θ=π when reflection occurs from more dense media, including massive defects. For θ=0 and θ=π, *cosine* and *sine* standing wave modes develop, respectively. In the first case, the motion at defect (or interface) location is maximized; in the second case, the motion at defect location is minimized. In case of a scattering resonance, there is an energy window (~ several Γ) where a sharp transition from θ=0 to θ=π takes place; and exactly at resonance θ=π/2. Generally, when the frequency of scattered waves is higher than the resonance frequency, π/2<θ<π and main contribution to standing waves is provided by s*ine* modes. The amplitude ratio of *cosine* and *sine* modes is estimated below.

When an incident wave e^{-ikx} is reflected with an additional phase shift θ, the interference field becomes: $e^{-ikx} + e^{ikx+i\theta} = e^{i\theta/2}\cos(kx + ½\theta) = \mathcal{A}_{\sin}\sin kx + \mathcal{A}_{\cos}\cos kx$.

We find that the amplitude ratio of *cosine* and *sine* modes inside a standing wave is $\mathcal{A}_{\cos}/\mathcal{A}_{\sin} = \cot(½\theta)$. Because for *cosine* modes, phonon superposition across the BZ yields $A(x) \propto x^{-1}\sin Kx$ dependence, which is undetectable at all atomic sites except for at x=0, interference of these modes only produces “zero” feature at defect location that can be observed in Fig. 3c of the manuscript.

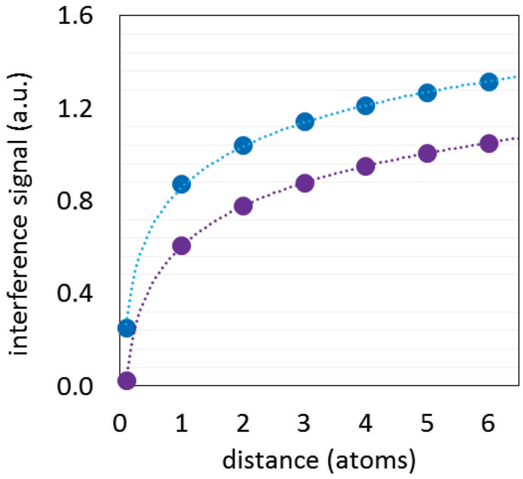

Supplementary Figure 1. Simulation of the background curve. For phonon scattering at defects, each phonon mode produces a separate contribution to standing wave pattern $A_k(x) \propto \sigma_k \cos(kx + \theta/2)$, where σ_k is k-dependent scattering probability, and θ is scattering phase shift. For coherent synchronized component of phonon oscillations, the total signal observed by STM can be obtained by linear summation of all A_k across the 2D BZ, which in case of $\sigma_k \propto k^{-1}$ yields essentially the same result as equation (1) of the manuscript. For incoherent (non-synchronized) phonon oscillation component, using such linear superposition is not correct because all phonon modes oscillate at different frequencies. However, the total average motion in this case can be estimated using quadratic superposition of all A_k : $\overline{A^2} = \sum A_k^2$. The simulation results, presented in the Figure, resemble the experimentally observed background curve in Fig. 5b of the manuscript. A broad central interference minimum represents the only feature produced by incoherent component. The purple circles correspond to θ=180°. The blue circles correspond to θ=162°. In principle, the background curve may include other than ZO_2 contributions (see Supplementary Ref. 1).

Supplementary Note 3. Discussion about phonon populations.

The possibility of population “pumping” by tunneling electrons can be ruled out. For typical currents, used in our STM measurements, electron tunneling occurs with an interval of ~5 ns, which is two orders of magnitude larger than the lifetime of ZO_2 phonons. The probability of inelastic tunneling is ~1%, which corresponds to excitation of << 1 ZO_2 phonon per electron tunneling event and is insufficient to generate a coherent phonon population. Another non-equilibrium mechanism, potentially affecting local phonon population, is phonon tunneling (see Supplementary Refs. 2-4) which may occur for significant STM tip vs. sample temperature difference. This mechanism was only reported for significantly shorter (2÷3 Å) tip-sample distances, corresponding to tunneling barrier resistance of 10^7 Ω. For tunneling resistance of ≈10^{11} Ω (used for all reported STM measurements), this mechanism can also be safely ruled out (Supplementary Ref. 2) even for significant STM tip vs. sample temperature difference.

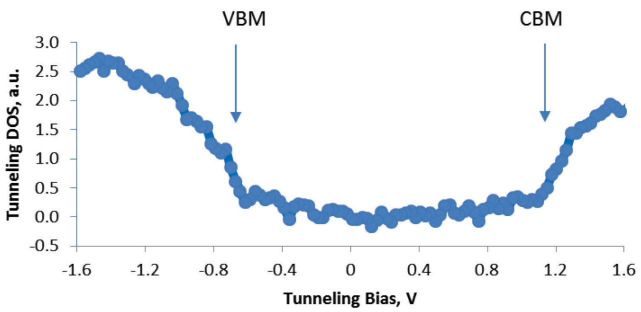

Supplementary Figure 2. Tunneling density of states for studied islands. Tunneling spectrum of quasi-freestanding 1 ML WSe_2 island measured near the defect location. The measurements were accomplished using 25 mV modulation at the frequency 1500 Hz. To compensate the exponential increase of signals at large bias (both positive and negative), the vacuum gap was slowly increased, at a rate of 0.4 Å per |V| as in Supplementary Ref. 4. For this spectrum, the band gap is 0.2 eV larger than photoluminescence studies of 1 ML WSe_2 earlier reported (Supplementary Ref. 5). The horizontal axis in this Figure is sample bias.

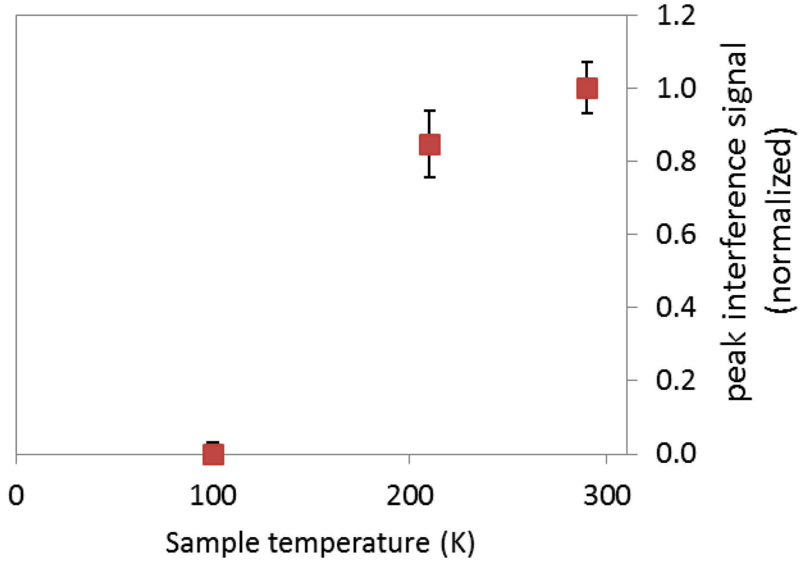

Supplementary Figure 3. Temperature dependence of interference signals. The temperature dependent heights of the first interference maxima are shown normalized on their average room-temperature value. Between room temperature and 210 K, the decrease of interference signals is comparable to anticipated decrease of phonon oscillation amplitudes. Between 210 K and 100 K, the decay of interference signals is more significant indicating a rapid decrease of the condensate fraction. At 100 K, only type-C patterns were observed.

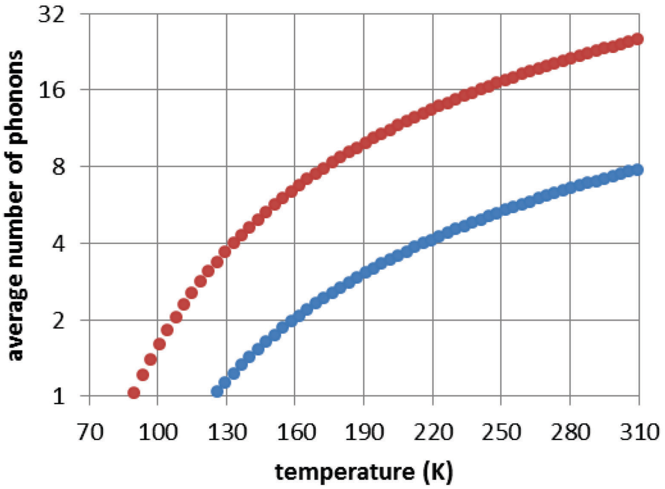

Supplementary Figure 4. Temperature dependence of average phonon numbers. Red and blue curves show the average number of thermal phonons inside $4.5a_0$ and $2.5a_0$ radius areas (estimated areas of type-B and type-A droplets). The phonon numbers are calculated using Planck's distribution with $\hbar\omega_{OP} = 360\ \mathrm{K}$. The estimated droplet sizes correspond to locations of 1st and 2nd interference minima.

Supplementary References

1. U. Rössler, Solid State Theory: An Introduction, Springer Science & Business Media, 2004

2. S. Xiong *et al.*, Phys. Rev. Lett. 112, 114301 (2014)

3. I. Altfeder, A. A. Voevodin, A. K. Roy, Phys. Rev. Lett. 105, 166101 (2010)

4. I. Altfeder, K. A. Matveev, A. A. Voevodin, Phys. Rev. Lett. 109, 166402 (2012)

5. P. Tonndorf *et al.*, Opt. Express 21, 4908 (2013)
